# Supplementary material for: Placental histopathology in preterm birth with confirmed maternal infection: A systematic literature review
Source: PLoS One. 2021 Aug 12;16(8):e0255902. doi: 10.1371/journal.pone.0255902 (PMC8360573; doi:10.1371/journal.pone.0255902)
Supplement: S1 Table — (DOCX) [file pone.0255902.s001.docx]

**S1 Table. Search terms used for systematic review**

| **Pubmed/Medline (1946 to present): 846 articles found**  (((((Pregnan*) AND ((preterm*) OR prematur*))) AND ((placenta*) AND (((patholog*) OR histopatholg*) OR histolog*)))) AND ((((infect*) OR microorganism*) OR bacteria*) OR virus*) OR parasite*))))) |
| --- |
| **Web of science (1900-present): 534 articles found**  ALL=(pregnan*) AND ALL=(preterm* OR premature*) AND ALL=(placenta*) AND ALL=(histolog* OR histopatholog* OR histolog*) AND ALL=(infect* OR microorganism* OR bacteria* OR virus* OR parasite*) |
| **Scopus (2004-present): 986 articles found**  ( ( ( TITLE-ABS-KEY ( pregnan* ) )  AND  ( TITLE-ABS-KEY ( preterm*  OR  premature* ) ) )  AND  ( ( TITLE-ABS-KEY ( placenta* ) )  AND  ( TITLE-ABS-KEY ( histolog*  OR  histopathog*  OR  histolog* ) ) ) )  AND  ( TITLE-ABS-KEY ( infect* OR  microorganism*  OR  bacteria*  OR  virus* OR parasite* ) ) |
| **Embase (1974-present): 911 articles found**  ALL=(pregnan*).mp. AND ALL=(preterm* OR premature*).mp. AND ALL=(placenta*).mp. AND ALL=(histolog* OR histopatholog* OR histolog*).mp. AND ALL=(infect* OR microorganism* OR bacteria* OR virus* Or parasite*).mp. |
