# Supplementary material for: Placental histopathology in preterm birth with confirmed maternal infection: A systematic literature review
Source: PLoS One. 2021 Aug 12;16(8):e0255902. doi: 10.1371/journal.pone.0255902 (PMC8360573; doi:10.1371/journal.pone.0255902)
Supplement: S2 Table — (DOCX) [file pone.0255902.s002.docx]

**S2 Table. Clinical characteristics associated with placental histopathology**

|  | maternal | | | | | | | | fetal | delivery | | | | | infection | | | | | |  |  | |
| --- | --- | --- | --- | --- | --- | --- | --- | --- | --- | --- | --- | --- | --- | --- | --- | --- | --- | --- | --- | --- | --- | --- | --- |
|  | Smoking / Drugs | History PTB (PROM) | BMI or Stature | Cervical incompetence | Gravidity / parity | Age / teenage | Preeclampsia / eclampsia | Medical complications e.g autoimmune | Fetal monitoring / fetal distress | Antenatal corticosteroid | Caesarean section *vs* vaginal birth | PTB spontaneous | PTB induced | Premature PROM | Study treatment | Number of VE | ANC screening for BV | ANC screening for GBS | Treatment for PROM / PPROM e,g Ampi/Pen | Length of ROM | Total (maximum score 20) | comment |  |
| **Bacteria** |  |  |  |  |  |  |  |  |  |  |  |  |  |  |  |  |  |  |  |  |  |  |  |
| Cox, 2016 [1] | 0 | 0 | 0 | 0 | 0 | 1 | 0 | 0 | 0 | 0 | 0 | 1 | 0 | 0 | 0 | 0 | 0 | 0 | 0 | 0 | 2 |  |  |
| Dammann, 2003 [2] | 0 | 0 | 0 | 0 | 0 | 0 | 1 | 0 | 0 | 1 | 1 | 1 | 0 | 1 | 0 | 0 | 0 | 0 | 1 | 1 | 7 | *U. urealyticum* culture routine if bw <1501g |  |
| Hecht, 2008[3] | 0 | 0 | 0 | 1 | 0 | 0 | 1 | 0 | 1 | 1 | 1 | 0 | 1 | 0 | 0 | 0 | 0 | 1 | 0 | 1 | 7 |  |  |
| Hillier, 1988 [4] | 0 | 0 | 0 | 0 | 0 | 1 | 0 | 0 | 0 | 0 | 0 | 1 | 0 | 1 | 1 | 0 | 0 | 0 | 1 | 1 | 6 |  |  |
| Hillier, 1991 [5] | 1 | 1 | 0 | 0 | 1 | 0 | 1 | 1 | 0 | 0 | 1 | 1 | 0 | 1 | 0 | 0 | 0 | 0 | 0 | 1 | 9 |  |  |
| Honma, 2007 [6] | 0 | 0 | 0 | 0 | 0 | 0 | 0 | 0 | 0 | 0 | 1 | 0 | 0 | 1 | 0 | 0 | 0 | 0 | 0 | 0 | 2 |  |  |
| Ingrid, 2011 [7] | 0 | 0 | 0 | 0 | 1 | 1 | 1 | 0 | 1 | 0 | 1 | 1 | 0 | 1 | 1 | 0 | 0 | 0 | 1 | 0 | 9 |  |  |
| Kwak, 2014 [8] | 0 | 0 | 0 | 1 | 1 | 1 | 1 | 1 | 0 | 0 | 0 | 1 | 1 | 1 | 0 | 0 | 0 | 0 | 0 | 0 | 7 |  |  |
| Namba, 2010 [9] | 0 | 0 | 0 | 0 | 0 | 0 | 1 | 0 | 0 | 0 | 0 | 1 | 1 | 0 | 1 | 0 | 0 | 0 | 0 | 0 | 4 |  |  |
| Patel, 2018 [10] | 1 | 0 | 0 | 0 | 0 | 1 | 0 | 0 | 1 | 1 | 0 | 0 | 0 | 1 | 1 | 0 | 0 | 0 | 1 | 1 | 8 |  |  |
| Pettker, 2007 [11] | 0 | 1 | 0 | 0 | 1 | 1 | 0 | 0 | 1 | 1 | 1 | 1 | 1 | 1 | 0 | 1^ | 0 | 0 | 1 | 1* | 12 | *interval amniocentisis to delivery in hours; ^ no VE in PPROM |  |
| Queiros da Mota, 2013 [12] | 0 | 0 | 0 | 0 | 0 | 0 | 0 | 0 | 0 | 0 | 1 | 1 | 0 | 1 | 1 | 0 | 0 | 0 | 1 | 0 | 5 |  |  |
| Sweeney 2016 [13] | 0 | 0 | 0 | 1 | 1 | 1 | 0 | 0 | 0 | 0 | 1 | 0 | 0 | 1 | 1 | 0 | 0 | 0 | 1 | 0 | 7 |  |  |
| **Virus** |  |  |  |  |  |  |  |  |  |  |  |  |  |  |  |  |  |  |  |  |  |  |  |
| Ategeka, 2019 [14] | 0 | 0 | 0 | 0 | 1 | 1 | 0 | 0 | 0 | 0 | 0 | 0 | 0 | 0 | 1 | 0 | 0 | 0 | 0 | 0 | 3 | All on ARV, Stage 1, RCT Cotri+Dp vs Cotri, p=0.07 |  |
| Feist, 2020 [15] | 0 | 0 | 0 | 0 | 1 | 1 | 1 | 1 | 0 | 0 | 0 | 0 | 0 | 1 | 0 | 0 | 0 | 0 | 0 | 1 | 6 |  |  |
| Gichangi, 1993 [16] | 0 | 0 | 0 | 0 | 0 | 0 | 0 | 0 | 0 | 0 | 0 | 1 | 0 | 0 | 0 | 0 | 0 | 0 | 0 | 0 | 1 |  |  |
| Ladner, 1998 [17] | 0 | 0 | 0 | 0 | 1 | 1 | 0 | 0 | 0 | 0 | 0 | 1 | 0 | 0 | 1 | 0 | 0 | 0 | 0 | 1 | 5 | Women treated for STI but not HIV |  |
| Ombimbo, 2019 [18] | 0 | 0 | 0 | 0 | 0 | 1 | 1 | 1 | 0 | 0 | 1 | 1 | 0 | 0 | 1 | 0 | 0 | 0 | 0 | 0 | 6 | All on ARV, no mention of Cotri |  |
| Tsekoura, 2010 [19] | 0 | 0 | 0 | 0 | 1 | 1 | 0 | 0 | 0 | 0 | 0 | 1 | 0 | 1 | 0 | 0 | 0 | 0 | 0 | 0 | 4 |  |  |
| **Parasite** |  |  |  |  |  |  |  |  |  |  |  |  |  |  |  |  |  |  |  |  |  |  |  |
| Ategeka, 2020 [20] | 0 | 0 | 0 | 0 | 1 | 1 | 0 | 1 | 0 | 0 | 0 | 1 | 0 | 0 | 1 | 0 | 0 | 0 | 0 | 0 | 5 |  |  |
| Kapisi, 2017 [21] | 0 | 0 | 0 | 0 | 1 | 1 | 0 | 1 | 0 | 0 | 0 | 1 | 0 | 0 | 1 | 0 | 0 | 0 | 0 | 0 | 5 |  |  |
| Lufele, 2017 [23] | 1 | 0 | 0 | 0 | 1 | 1 | 0 | 0 | 0 | 0 | 0 | 1 | 0 | 0 | 1 | 0 | 0 | 0 | 0 | 0 | 5 | IPTp (SP+Az) or curative dose (SP+CQ) |  |
| Saad, 2017 [22] | 0 | 0 | 0 | 0 | 1 | 1 | 1 | 1 | 0 | 0 | 0 | 1 | 0 | 0 | 0 | 0 | 0 | 0 | 0 | 0 | 5 |  |  |

Abbreviations: Ampi Ampicillin; ANC antenatal care; AROM artificial rupture of membranes; ARV antiretroviral; Az azithromycin; BV bacterial vaginosis; Cotri cotrimoxazole; CQ chloroquine; DP dihydroartemisinin-piperaquine; GBS group B streptococcus; HIV human immunodeficiency virus; IPTp intermittent preventive treatment pregnancy; Pen Penicillin; PROM, preterm rupture of membranes; PPROM prolonged preterm rupture of membranes; PT preterm; PTB preterm birth; RCT randomized controlled trial; ROM rupture of membranes; SP sulphadoxine-pyrimethamine; STI sexually transmitted infection; VE vaginal examination
