# Supplementary material for: Placental histopathology in preterm birth with confirmed maternal infection: A systematic literature review
Source: PLoS One. 2021 Aug 12;16(8):e0255902. doi: 10.1371/journal.pone.0255902 (PMC8360573; doi:10.1371/journal.pone.0255902)
Supplement: S3 Table — (DOCX) [file pone.0255902.s003.docx]

**S3A Table. Risk of bias assessment using Newcastle – Ottawa Scale (NOS): Cohort studies.**

| **Author, Year, Reference** | **Selection** | | | | **Comparability** | | **Outcome** | | | **Total Stars** |
| --- | --- | --- | --- | --- | --- | --- | --- | --- | --- | --- |
|  | Representativeness | Selection of non-exposed cohort | Ascertainment of exposure | Outcome of interest was not present at start of study | Most important clinical characteristics | Additional characteristics | Assessment of outcome | Was follow-up long enough for outcomes | Adequacy of follow up of cohorts |  |
| Ategeka, 2019 ^$^ | * | * | * | * |  | * | * | NA | NA | 6 |
| Ategeka, 2020 ^$^ | * | * | * | * |  | * | * | NA | NA | 6 |
| Feist, 2020 | * | * | * |  |  | * | * | NA | NA | 5 |
| Hecht, 2008 | * | * | * | * | * | * | * | NA | NA | 7 |
| Honma, 2007 | * | * | * | * |  |  | * | NA | NA | 5 |
| Ingrid, 2011 | * | * | * | * | * | * | * | NA | NA | 7 |
| Kapisi, 2017 ^$^ | * | * | * | * |  | * | * | NA | NA | 6 |
| Kwak, 2014 | * | * | * | * | * | * | * | NA | NA | 7 |
| Ladner, 1998 | * | * | * | * |  | * | * | NA | NA | 6 |
| Lufele, 2017 | * | * | * | * |  | * | * | NA | NA | 6 |
| Ombinbo, 2019 | * | * | * | * |  | * | * | NA | NA | 6 |
| Namba, 2010 | * | * | * | * |  |  | * | NA | NA | 5 |
| Patel, 2018 | * | * | * | * | * |  | * | NA | NA | 6 |
| Querios da Mota, 2013 | * | * | * | * |  | * | * | NA | NA | 6 |
| Saad, 2017 | * | * | * | * |  | * | * | NA | NA | 6 |
| Sweeney, 2016 | * | * | * | * |  | * | * | NA | NA | 6 |

$: Sub-analysis of randomized control trial of malaria prevention studies. *Star: The NOS tool uses a star system to assess the methodological quality based on three criteria; i) participants’ selection (4 stars), ii) comparability of study groups (2 stars), and iii) assessment of outcome/exposure (3 stars). Therefore, the highest total score for a cohort study was 7. Follow-up long enough for outcomes and adequacy of follow-up of cohorts are not applicable in these reviewed articles. NA: Not applicable.

**S3B Table. Risk of bias assessment using Newcastle – Ottawa Scale (NOS): Case-Control studies.**

| **Author, Year, Reference** | **Selection** | | | | **Comparability** | | **Exposure** | | | **Total Stars** |
| --- | --- | --- | --- | --- | --- | --- | --- | --- | --- | --- |
|  | Case definition | Representativeness | Selection of control | Definition of control | Most important clinical factors | Additional factors | Ascertainment of exposure | Method of ascertainment | Non-Response rate |  |
| Cox, 2016 | * | * | * | * |  |  | * | * | NA | 6 |
| Dammann, 2003 | * | * | * | * | * | * | * | * | NA | 8 |
| Gichangi, 1993 | * | * | * | * |  |  | * | * | NA | 6 |
| Hillier, 1988 | * | * | * | * |  | * | * | * | NA | 7 |
| Hillier, 1991 | * | * | * | * | * | * | * | * | NA | 8 |
| Pettker, 2007 | * | * | * | * | * | * | * | * | NA | 8 |
| Tsekura, 2010 | * | * | * | * |  |  | * | * | NA | 6 |

*Star: The NOS tool uses a star system to assess the methodological quality based on three criteria; i) participants’ selection (4 stars), ii) comparability of study groups (2 stars), and iii) exposure (2 stars). Therefore, the highest total score for a case-control study was 8. Follow-up long enough for outcomes and adequacy of follow-up of cohorts are not applicable in these reviewed articles. NA: Not applicable
