# Supplementary material for: Placental histopathology in preterm birth with confirmed maternal infection: A systematic literature review
Source: PLoS One. 2021 Aug 12;16(8):e0255902. doi: 10.1371/journal.pone.0255902 (PMC8360573; doi:10.1371/journal.pone.0255902)
Supplement: S4 Table — (DOCX) [file pone.0255902.s004.docx]

**S4A Table. Placental histopathological sample collection and classification system**

| **Author name, Year*** | **Details of sample collection** | **Classification system used for placental histopathology** |
| --- | --- | --- |
| Cox, 2016 ^26^ | “Tissue samples were collected from the sub-chorionic plate area on the fetal side of the placenta” | Kraus, 2004 ^60^ categorized the histopathological samples as the presence or absence of chorioamnionitis by PMN leucocytes.  Severity of HP changes-not done |
| Dammann, 2003 ^27^ | “Formalin-fixed, paraffin-embedded, haematoxylin and eosin-stained sections of the umbilical cord, placental membranes and placental parenchyma.” | Kaplan, 1991 ^58^ based presence of chorioamnionitis on the report by the College of American pathologists conference XIX.  Severity of HP changes-not done. |
| Hecht, 2008 ^31^ | “Sections of the umbilical cord and a membrane roll and full-thickness sections from the center and a paracentral zone of the placental disc.” | Driscoll SG, 1991 ^54^ defined histologic chorioamnionitis based on the report by the College of American pathologists conference XIX, while inflammation of the membrane using the scoring system similar to that proposed by *Redline, 2003 ^10^  Severity of HP changes-done and used for comparison. |
| Hillier, 1988 ^33^ | “The fetal membranes (chorioamnion) were grasped with forceps, and a strip 2 to 3 cm wide was cut from the point of rupture to the placental margin. A “membrane role” was made by rolling the fetal membranes onto a 1-mm wooden dowel. The membrane was fixed in 10 percent neutral buffered formalin, imbedded in paraffin, and stained with hematatoxylin and eosin.” | Author own: “≧ 10 PMN leucocyte per 10 non-adjacent field (x400 power)”  Severity of HP changes-not done. |
| Hillier, 1991 ^32^ | “The fetal membranes were grasped with forceps, and a 2 to 3 cm strip was cut from the point of rupture to the discoid placental margin to prepare a "membrane roll" for histologic examination.” | Author own: “≧ 10 PMN leucocyte per 10 non-adjacent field (x400 power).”  Severity of HP changes-not done. |
| Honma, 2007 ^34^ | Not stated | Author own: “PMN leucocyte detected in chorion, amnion, subchorion and funisitis if PMN leucocytes found in umbilical vessels wall.”  Severity of HP changes-not done. |
| Ingrid, 2011 ^35^ | “Immediately after delivery, placentas and membranes were fixed in formalin for at least 6 hours. Sampling was performed with two membrane rolls, two cross sections of the cord and three representative blocks of the placental disk as a minimum. The tissues were embedded in paraffin until histopathological examination.” | *Redline, 2003 ^10^ was used to categorized the placental inflammation according to 3 stages of maternal inflammatory response and /or fetal inflammatory response.  Severity of HP changes- done and used for comparism. |
| Kwak, 2014 ^37^ | Not stated | Author own: “presence of acute inflammatory changes of membranes of the placenta after delivery”  Severity of HP changes-not done. |
| Namba, 2010 ^40^ | Not stated | Blanc, 1981 ^65^  Severity of HP changes- done. |
| Patel, 2018 ^42^ | Not stated | **Khong, 2016 ^59^  Severity of HP changes-not done. |
| Pettker, 2007 ^43^ | “An approximately 1 cm^2^ portion of amnion-chorion taken at a location away from the site of membrane rupture (amnion–chorion biopsy), and an approximately 1 cm^3^ partial-thickness section from the fetal side of the placenta (placenta biopsy).” | Author own (modified): “Three histologic stages of chorioamnionitis (stage I: intervillositis, stage II: chorionic inflammation, and stage III: full-thickness inflammation of both chorion and amnion) were complemented by the histologic grading system devised by Salafia *et al.* ^67^, which includes four grades of inflammation of the amnion, chorion– decidua, and umbilical cord.”  Severity of HP changes-done and used for comparison. |
| Queiros da Mota, 2013 ^44^ | “Each placenta was fixed in 10% buffered formalin solution for 48 h. After examination of the appearance, colour and insertion of the amniotic membranes, a 10 cm-wide membrane roll was rolled with the amnion inside, from the rupture site to the placental margin. Samples were taken at two levels of the roll, each containing the rupture site and the placental margin. Three samples were taken (at the two extremities and in the middle). The placental disk was measured and weighed after removal of the membranes and cord, and the fetal and maternal surfaces examined. Placental tissue was sampled, each 5–10 mm, including the area at the cord insertion, and at least three full-thickness villous tissue samples. All samples were less than 1 cm thick. Microscopic examination was performed using H&E-stained sections.” | Kalousek, 1992 ^57^  Severity of HP changes-done and used for comparison. |
| Sweeney 2016 ^45^ | Placentae were refrigerated under strict aseptic conditions within 24 hours of delivery. The external placental surface was decontaminated using 70% alcohol, and areas where the amnion had detached from the placenta were avoided, to minimize contamination. An incision was made into the amnion (ie, uppermost) membrane, and the interface between the amnion and chorion was identified. Chorioamnion tissue was also excised and placed into sterile cryogenic vials. All samples were stored at −80°C prior to analysis. | *Redline, 2003 ^10^  Severity of HP changes-done and used for comparison |
| Ategeka, 2019 ^25^ | “All placental sampling was done within 30 minutes of delivery. Placental specimens were fixed in 10% Neutral Buffered Formalin for 24 hours, and stored at room temperature in 70% ethanol prior to tissue processing. Collected tissue included: placental membranes (“membrane roll”), umbilical cord (two cross-sectional slices, one proximal and one distal to where the cord inserts into the placental disc), and chorionic plate with villous parenchyma. Specimens were dehydrated through a series of ethanol washes, cleared in xylene and embedded in paraffin wax blocks. A 3 μm thick section from each tissue block was obtained using a rotary microtome and sections mounted onto glass slides via a floatation water bath. Slides were baked in a hot air oven at 60˚C for 30 minutes, de-paraffinized in xylene, dehydrated through a series of ethanol washes, stained with Hematoxylin and Eosin (H&E), and mounted with organic mounting media.” | *Redline, 2003 ^10^  Severity of HP changes-not done. |
| Feist, 2020 ^28^ | “At least two paraffin blocks of the umbilical cord tissue and the fetal membranes and at least four blocks from the placental parenchyma were produced for histological investigations.” | **Khong, 2016 ^59^ and Baergen RN, 2011 ^52^ were used to classify the histological lesions.  Severity of HP changes- done (high and low grade) and not used for comparison. |
| Gichangi, 1993 ^29^ | “Placenta obtained at delivery were fixed in 10% formalin for a period of two weeks to one month. Free membranes, umbilical cord and placenta were processed.” | Placental leucocytic infiltration was reported as 0=no infiltration, 1= less than 5 PMN, 2= 2-5 PMN and 3= more than 10 PMN on high power field microscopy as described by Benirschke, 1961 ^53^.  Severity of HP changes-not done. |
| Ladner, 1998 ^38^ | “Tissues from membrane and placenta were fixed in 10% formalin and stained with haemtoxylin and eosin. Tissues from membranes and placenta..” | Author own: “≧ 10 PMN leucocyte in the amnion, chorioamniotic plate or both per several non-adjacent x400 power fields”  Severity of HP changes-not done. |
| Ombimbo, 2019 ^41^ | Six biopsies of the placentas and fetal membranes were obtained immediately after delivery (2 central from either side of the cord insertion and 4 from peripheral aspects of the placenta at the 12, 3, 6, and 9 o’clock positions) and fixed in 10% neutral buffered formalin. The membranes were removed from the placentas and made into rolls; the rolls and placental biopsies were dehydrated in increasing concentrations of alcohol (70% -100%), 1 hour per solution. Then, they were cleared in trichloroethane (TCE) for 2 hours and infiltrated with paraffin for 12 hours. Finally, the specimens were embedded in fresh molten paraffin for 12 hours overnight. Blocks, which were sectioned and stained. Five micrometer serial sections were cut using a Leitz Wetzlar sledge microtome, floated in warm water, mounted on glass slides, and dried in a hot air oven at 40°C overnight. The sections were stained with Masson’s trichrome, picrosirius red, or hematoxylin and eosin (H&E). H&E was used to demonstrate the general histoarchitecture. Masson’s trichrome highlighted the connective tissue components. Picrosirius red stained collagen fibers. | Hromatka, 2013 ^56^ was used to score the other features of placenta changes such as villus degeneration, syncytiotrophoblast delamination, vascularity, red blood cell adhesion to terminal villi, intermediate mature to mature villi, syncytial knotting, villitis, and deciduitis.  Terminal villi were scored using the method described in Altshuler, 1984 ^51^.  Severity of HP changes-not done. |
| Tsekoura, 2010 ^46^ | “Samples from the peripheral membranes stained with routine hematoxylin-eosin were blinded and evaluated by light microscopy to diagnose histological chorioamnionitis.” | Author own “The presence of neutrophils in the membranes (chorion and/or amnion) was used to define chorioamnionitis.”  Severity of HP changes-not done. |
| Ategeka, 2020 ^24^ | “The basal plate was trimmed into a 3-mm slice and dehydrated through a series of ethanol washes, cleared in xylene, and embedded in paraffin wax blocks. A 3-μm thick section from each tissue block was obtained using a rotary microtome, and sections were mounted onto glass slides via a flotation water bath. Slides were baked in a hot air oven at 60°C for 30 minutes, deparaffinized in xylene, dehydrated through a series of ethanol washes, stained with hematoxylin and eosin, and mounted with organic media. The histopathological process underwent extensive quality assurance to eliminate formalin pigment and minimize the effect of other artifacts. | Bulmer, 1993 ^23^ defined as uninfected (no evidence of parasites or pigment), active infection (parasites detected, no malaria pigment in fibrin), active-chronic (parasites detected and malaria pigment in fibrin), or past-chronic (parasites not detected, malaria pigment in fibrin).  Muehlenbachs, 2010 ^61^ describes a simplified score that for grading the histologic features of placental malaria (PM). *A*, Categories of maternal inflammation in the intervillous spaces: I, minimal; II, present; and III, massive. *B*, Categorizing malarial pigment deposition in intervillous fibrin.  Severity of HP changes-done and not used for comparison. |
| Kapisi, 2017 ^36^ | “Collection of specimens within 1 h of delivery, including placental tissue. Formalin-fixed paraffin-embedded placental biopsies were processed in duplicate for histological evidence of placental malaria.” | First the reader is referred to Kakuru, 2016 ^68^ which then refers the reader to two manuscripts. The first is Rogerson, 2003 ^63^ with 5 categories: 1. Not infected - No malaria parasites or pigment, 2. Past infection- No parasite, only pigment, 3. Parasites present and pigment in fibrin, 4. Parasites, pigment in monocyte ± fibrin, 5. Parasite, no pigment in monocyte or fibrin.  Muehlenbachs, 2010 ^61^ as described above.  Severity of HP changes-not done. |
| Lufele, 2017 ^39^ | Placental biopsies were collected from the maternal side of the placenta. Incisions extended from the maternal to the fetal side of the placenta without reaching the fetal membrane. Biopsies were fixed and transported in 10% neutral buffered formalin. Sections were stained with Giemsa, cover slipped and returned to PNG for analyses. | Ismail, 2000 ^22^  Rogerson, 2003 ^63^ as described above.  Severity of HP changes-not done. |
| Saad, 2017 ^30^ | “The placenta was collected in a clean labelled container and was preserved in 10% formaldehyde. After 24 hours of 10% formaldehyde fixation, the coded samples were processed for routine paraffin embedding. Multiple sections of 4 μm thickness were cut on a rotary microtome from the middle of each specimen and mounted on clean gelatinized slides for H&E staining.” Ten fields in 4 examined slides (ie total of 40 fields) were examined for each sample. In each field; the 10 smallest terminal villi (less than 80 μm in diameter) were microscopically evaluated, and then the mean was calculated.” | Tantbirojn, 2009 ^64^ was used to classify the inflammatory changes, infarction, intervillous thrombosis, chorionic villitis, haemorrhagic endovasculitis, placental intravascular thrombi, trophoblast degenerative knots, perivillous fibrin deposition and fibrinoid necrosis and villous oedema.  Both Gordijn, 2008 ^55^ and Khong, 2003 ^66^ were referenced to support scoring systems reported in “Quality of placental pathology reports” by giving 1 point to each of the previous criteria. The mean score was finally calculated for each group.  Severity of HP changes-not done. |

Abbreviations: PMN; polymorphonuclear

^*^see Redline 2003 detailed in S4B Table (below)

^**^see Khong 2016 detailed in S4C Table (below)

**S4B Table. Redline *et al.*, 2003: nomenclature and definitions of placenta reaction patterns related to amniotic fluid infection ^10^**

| **Diagnostic categories** | **Suggested diagnostic terminology** | **Definitions** |
| --- | --- | --- |
| **Maternal inflammatory response** | | |
| **Stage** |  |  |
| 1—Early | Acute subchorionitis or chorionitis | PMN in subchorionic fibrin and/or membrane trophoblast |
| 2—Intermediate | Acute chorioamnionitis | Diffuse-patchy PMN in fibrous chorion and/or amnion |
| 3—Advanced | Necrotizing chorioamnionitis | PMN karyorrhexis, amniocyte necrosis, and/or amnion basement membrane thickening/hypereosinophilia |
| **Grade** |  |  |
| 1—Mild–moderate | No special terminology required | Not severe as defined below |
| 2—Severe | Severe acute chorioamnionitis or with subchorionic micro-abscesses | Confluent PMN (≥ 10 x 20 cells in extent) between chorion and decidua; ≥ 3 isolated foci or continuous band |
| Other | Chronic (or subacute) chorioamnionitis | Sub-amnionic mononuclear cell infiltrate with occasional PMN (meconium and hemosiderin-laden macrophages excluded) |
| **Fetal inflammatory response** | | |
| **Stage** |  |  |
| 1—Early | With chorionic vasculitis or umbilical phlebitis | Intramural PMN-chorionic vessels and/or umbilical vein |
| 2—Intermediate | With umbilical vasculitis (one or two arteries and vein) or umbilical panvasculitis (all vessels) | Intramural PMN-umbilical artery or arteries (umbilical vein) |
| 3—Advanced | With (subacute) necrotizing funisitis or with concentric umbilical perivasculitis | PMN associated debris in concentric bands-rings-halos around one or more umbilical vessels |
| **Grade** |  |  |
| 1—Mild–moderate | No special terminology required | Not severe as defined below |
| 2—Severe | With a severe fetal inflammatory response or with intense chorionic (umbilical) vasculitis | Near confluent intramural PMN-chorionic and/or umbilical vessels with attenuation/degeneration of VSMC |
| Other | With associated fetal vessel thrombi | Recent thrombosis associated with intramural PMN |
| Other specific features | Peripheral funisitis  Acute villitis  Acute intervillositis with intervillous abscesses  Decidual plasma cells | Focal aggregates of PMN at the umbilical cord surface  PMN in villous stroma (or between trophoblast and stroma)  Patchy-diffuse PMN in intervillous space  Unequivocal plasma cells in decidua basalis or capsularis |

PMN; polymorphonuclear leucocyte, VSMC; vascular smooth muscle cell

**S4C Table. Khong *et al.,* 2016: Staging and Grading of the Maternal and Fetal Inflammatory Responses in Ascending Intrauterine Infection by Amsterdam placental workshop group consensus 2016 ^56^**

| **Maternal Inflammatory Response** | |
| --- | --- |
| **Stage** | **Grade** |
| Stage 1—acute subchorionitis or chorionitis | Grade 1—not severe as defined |
| Stage 2—acute chorioamnionitis: polymorphonuclear leukocytes  extend into fibrous chorion and/or amnion | Grade 2—severe: confluent polymorphonuclear leukocytes or with subchorionic microabscesses |
| Stage 3—necrotizing chorioamnionitis: karyorrhexis of polymorphonuclear leukocytes, amniocyte necrosis, and/or amnion basement membrane hypereosinophilia |  |
| **Fetal Inflammatory Response** | |
| Stage 1—chorionic vasculitis or umbilical phlebitis | Grade 1—not severe as defined |
| Stage 2—involvement of the umbilical vein and one or more umbilical arteries | Grade 2—severe: near-confluent intramural polymorphonuclear  leukocytes with attenuation of vascular smooth muscle |
| Stage 3—necrotizing funisitis |  |
